# Author Correction: Division of labour between Myc and G1 cyclins in cell cycle commitment and pace control

**DOI:** 10.1038/s41467-018-07169-y

**Published:** 2018-11-13

**Authors:** Peng Dong, Manoj V. Maddali, Jaydeep K. Srimani, François Thélot, Joseph R. Nevins, Bernard Mathey-Prevot, Lingchong You

**Affiliations:** 1 Computational Biology and Bioinformatics Program, Duke University, Durham, North Carolina 27708 USA; 2 Department of Biomedical Engineering, Duke University, Durham, North Carolina 27708 USA; 3 Department of Pharmacology and Cancer Biology, Duke University, Durham, North Carolina 27708 USA; 4 Department of Molecular Genetics and Microbiology, Duke University, Durham, North Carolina 27708 USA; 5 Department of Pediatrics, Duke University, Durham, North Carolina 27708 USA; 6 Center for Genomic and Computational Biology, Duke University, Durham, North Carolina 27708 USA; 7 Duke Center for Systems Biology, Duke University, Durham, North Carolina 27708 USA; 8 Present Address: School of Medicine, Johns Hopkins University, Baltimore, Maryland 21205 USA

Correction to: *Nature Communications* 10.1038/ncomms5750; published online 01 September 2014

This Article contains errors in Supplementary Table 3. The sixth equation in the table should read:

$$\frac{{{d}[RB]}}{{{dt}}} \hskip 3pt = k_{{\mathrm{RB}}} + k_{{\mathrm{RBDP}}} \cdot \frac{{[RP]}}{{K_{{\mathrm{RP}}} + [RP]}} - k_{{\mathrm{RE}}} \cdot [RB] \cdot [E2Fp] - k_{{\mathrm{RBP1}}} \cdot \frac{{[CD] \cdot [RB]}}{{K_{{\mathrm{CD}}} + [RB]}} - k_{{\mathrm{RBP2}}} \cdot \frac{{[CE] \cdot [RB]}}{{K_{{\mathrm{CE}}} + [RB]}} - d_{{\mathrm{RB}}} \cdot [RB]$$

The simulation results in the Article were based on the correct formula and thus the results are not affected by this correction. The errors have not been fixed in the original Article.

